# Massive Mixed Adenoneuroendocrine Carcinoma: A Case Report

**DOI:** 10.7759/cureus.15928

**Published:** 2021-06-25

**Authors:** Christopher Millet, Alisa Farokhian, Nader Mekheal, Balraj Singh, Walid Baddoura

**Affiliations:** 1 Internal Medicine, St. Joseph's Regional Medical Center, Paterson, USA; 2 Hematology/Oncology, St. Joseph's University Medical Center, Paterson, USA; 3 Gastroenterology, St. Joseph's Regional Medical Center, Paterson, USA

**Keywords:** manec tumor, collision tumors, adenocarcinoma, mixed tumor, neuroendocrine carcinoma, mixed adenoneuroendocrine carcinoma

## Abstract

Mixed adenoneuroendocrine carcinoma (MANEC) of the gastrointestinal (GI) tract is a rare subtype of mixed tumors, and it is scarcely described in the literature. MANEC tumors are composed of adenocarcinoma and neuroendocrine carcinoma components, each of which comprises at least 30% of the lesion. Diagnosing MANEC requires specific histological and immunohistochemistry (IHC) analysis. Typically, MANEC tumors carry a poor prognosis due to their very aggressive nature.

We report the case of a 70-year-old female patient with no past medical history who presented with a three-week history of abdominal pain and one episode of hematemesis one week prior to presentation. Initial CT of the abdomen showed a large, 8 x 6 x 6-cm mass arising from the stomach and extending to the lesser sac as well as the central crus of the diaphragm with bilateral retroperitoneal lymphadenopathy. Upper endoscopy revealed an excavated, ulcerated, and partially necrotic mass on the lesser curvature of the proximal gastric body. Tissue biopsy of the lesion showed infiltrating mixed poorly differentiated adenocarcinoma and neuroendocrine carcinoma. On IHC, the adenocarcinoma component stained positively for CDX2 and pancytokeratin, and the neuroendocrine component stained positively for synaptophysin and chromogranin. Further workup included CT of the chest, which demonstrated extensive bilateral pulmonary emboli and new liver lesions with moderate ascites not seen on the initial abdominal CT. The latter was repeated and showed remarkable enlargement of the gastric mass (up to 12 cm) with extensive retroperitoneal adenopathy and mesenteric implants. Given the rapid clinical deterioration and progression of tumor burden, comfort measures were offered and the patient passed away soon after. MANEC tumors are highly aggressive subtypes of "collision" tumors, which are not well described in the medical literature due to their rarity. The etiology is poorly understood with various theories proposing different pathophysiological mechanisms. Standard therapy is not well developed at present; however, a few reports have demonstrated successful outcomes with surgery or combined chemotherapy (cisplatin with irinotecan or etoposide) if diagnosed at an early stage.

## Introduction

Mixed adenoneuroendocrine carcinoma (MANEC) of the gastrointestinal (GI) tract is a rare subtype of mixed tumors, and there is scarce data on them in the medical literature [1]. MANEC tumors, as the name implies, are composed of adenocarcinoma and neuroendocrine carcinoma components [1]. MANEC tumors are further classified into different types according to their histological characteristics. Classifying a neoplasm as MANEC requires specific histological and immunohistostaining analysis. Histological analysis of the neoplasm must show the adenoma/adenocarcinoma and neuroendocrine carcinoma cells each composing at least 30% of the lesion. The tissue biopsy must then undergo immunohistological staining with synaptophysin, chromogranin, and CD56. The diagnosis of MANEC is based on the presence of at least two of the three markers, along with the histologic criteria as described above. In this report, we discuss a case of MANEC tumor in the gastric body [2,3].

## Case presentation

**Figure 1:**
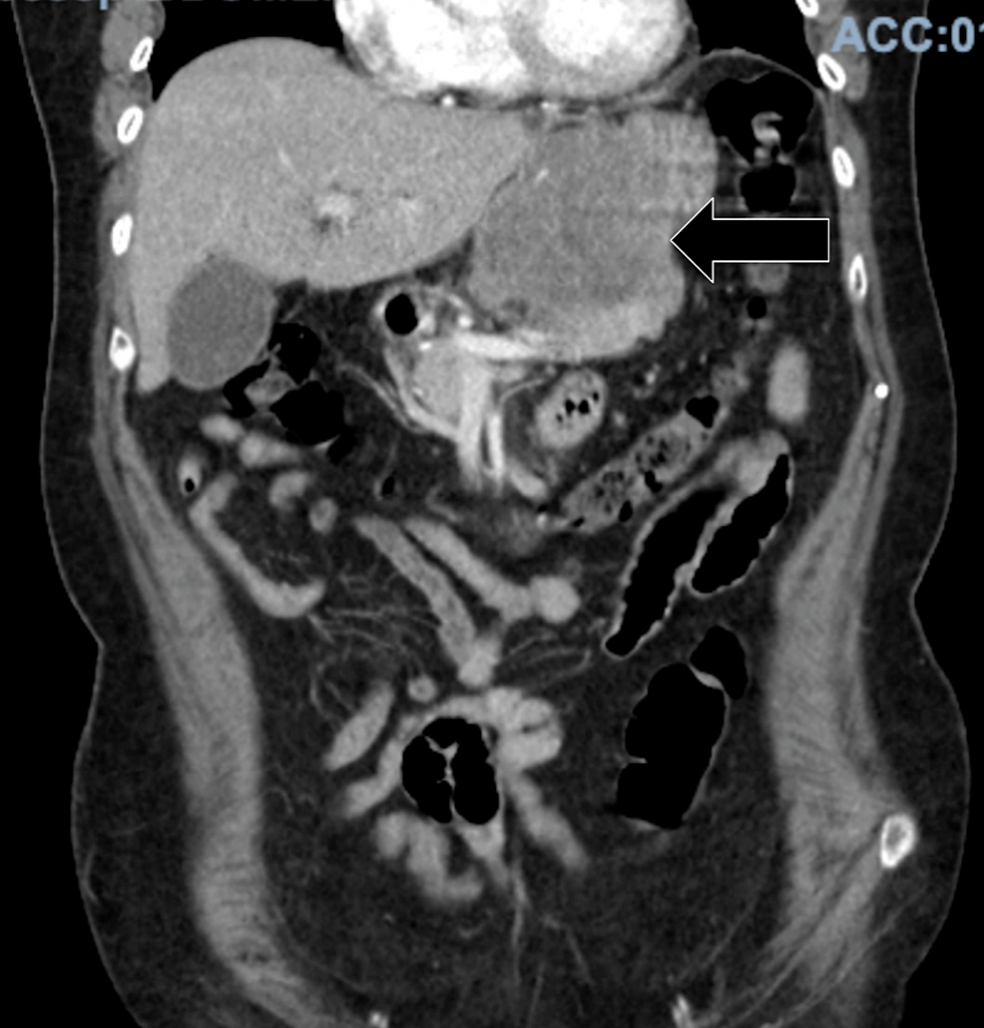
CT scan of the abdomen performed upon admission The image shows a large, 7.6 x 5.9 x 6.2-cm intraabdominal mass arising from the stomach and extending to the lesser sac and the central crus of the diaphragm (arrow) CT: computed tomography

**Figure 2:**
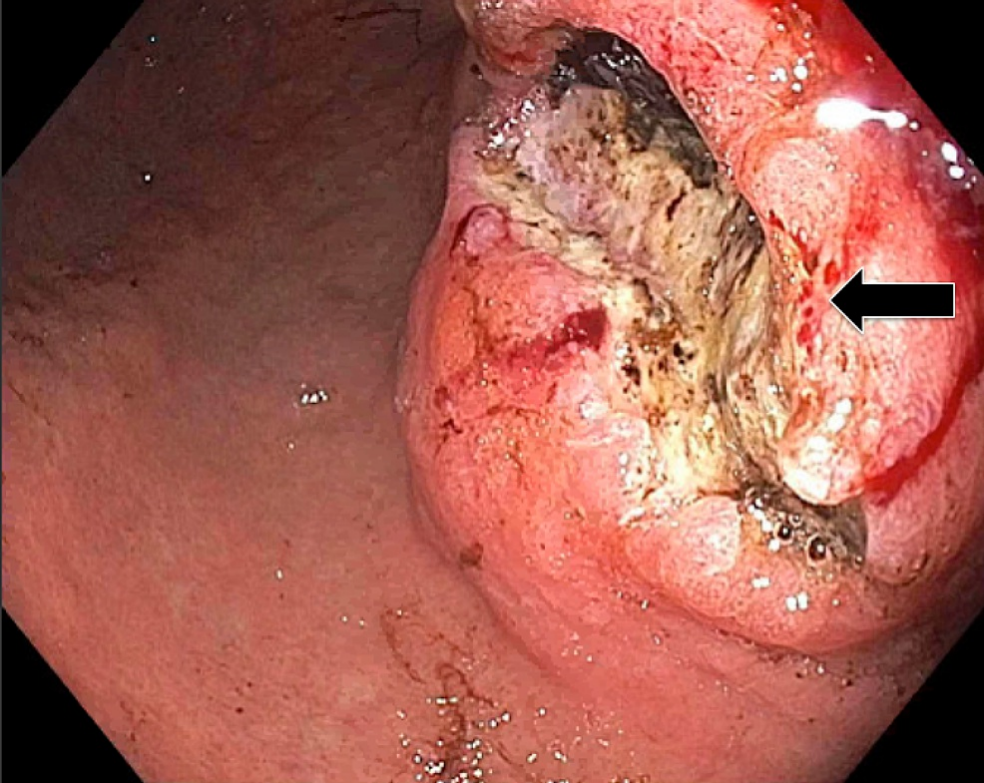
Esophagogastroduodenoscopy finding The image shows a large excavated, ulcerated, and partially necrotic mass with no bleeding on the lesser curvature of the proximal gastric body (arrow)

The patient was a 70-year-old female with no past medical history who presented with complaints of abdominal pain of three weeks' duration and one episode of hematemesis in the past week. At the emergency department, the patient underwent a CT scan of the abdomen, which exhibited a large, 8 x 6 x 6-cm abdominal mass arising from the stomach and extending to the lesser sac and central crus of the diaphragm with bilateral retroperitoneal lymphadenopathy (Figure 1). Admission labs were remarkable for a hemoglobin of 11.8 g/dl (normal range: 13.5-17.5), hematocrit of 34.6% (normal range: 41-53), alkaline phosphatase of 109 U/L (normal range: 34-104), and a sodium of 129 mEq/L (normal range: 135-145). Gastroenterology was consulted, and they performed an esophagogastroduodenoscopy (EGD), which revealed a large excavated, ulcerated, and partially necrotic mass on the lesser curvature of the proximal gastric body (Figure 2).

**Figure 3:**
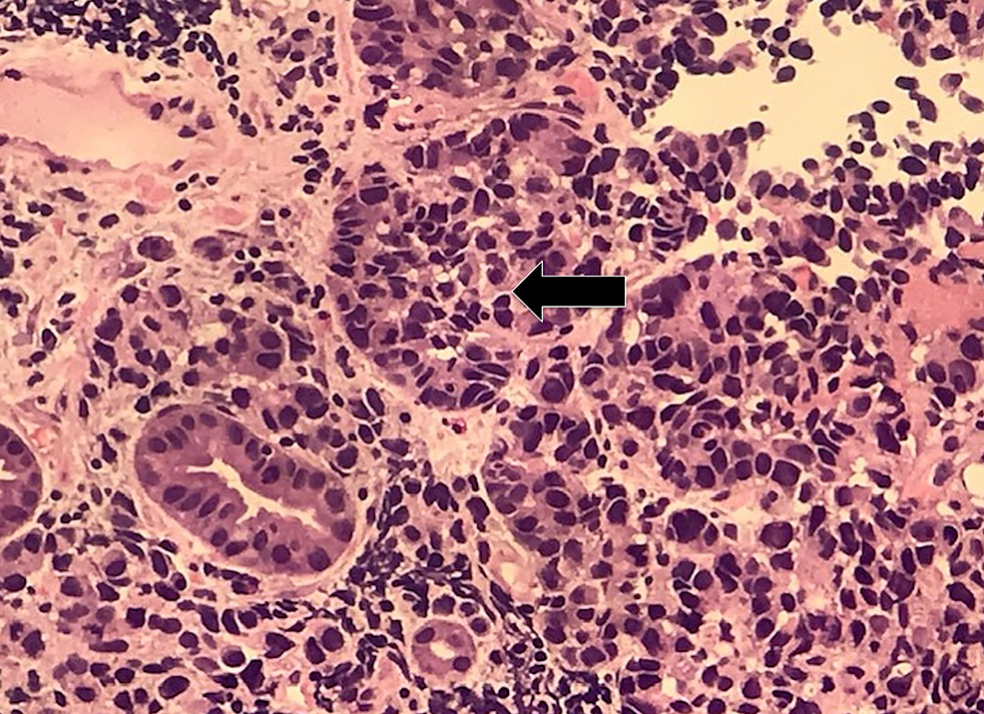
Poorly differentiated adenocarcinoma (arrow) (200x magnification)

**Figure 4:**
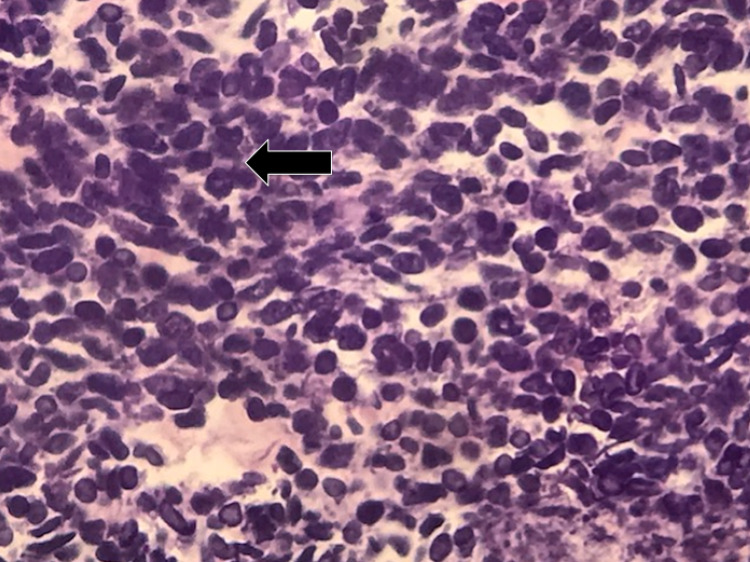
Neuroendocrine carcinoma (arrow) (200x magnification)

**Figure 5:**
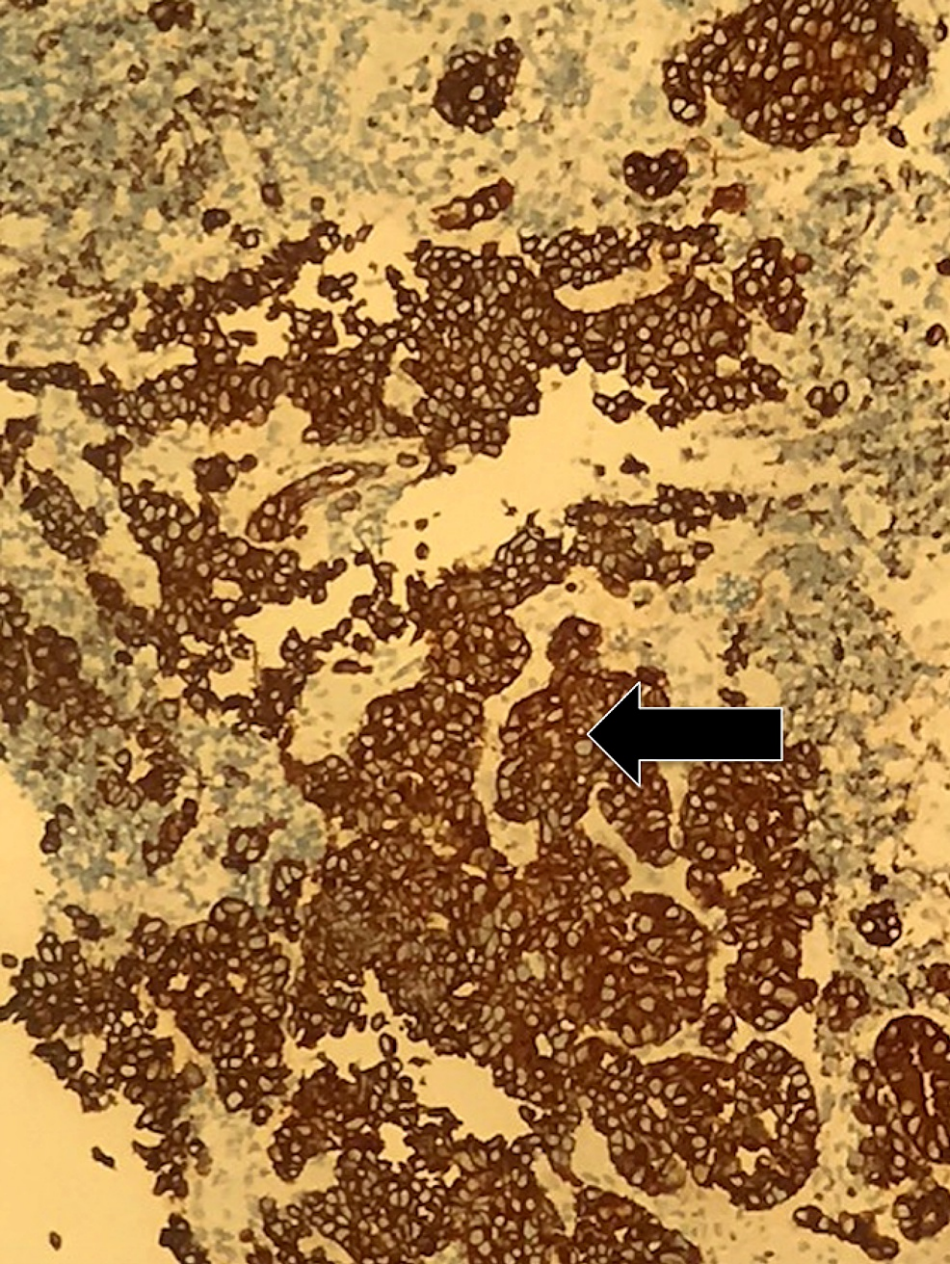
Pancytokeratin diffuse positivity in adenocarcinoma (arrow)

**Figure 6:**
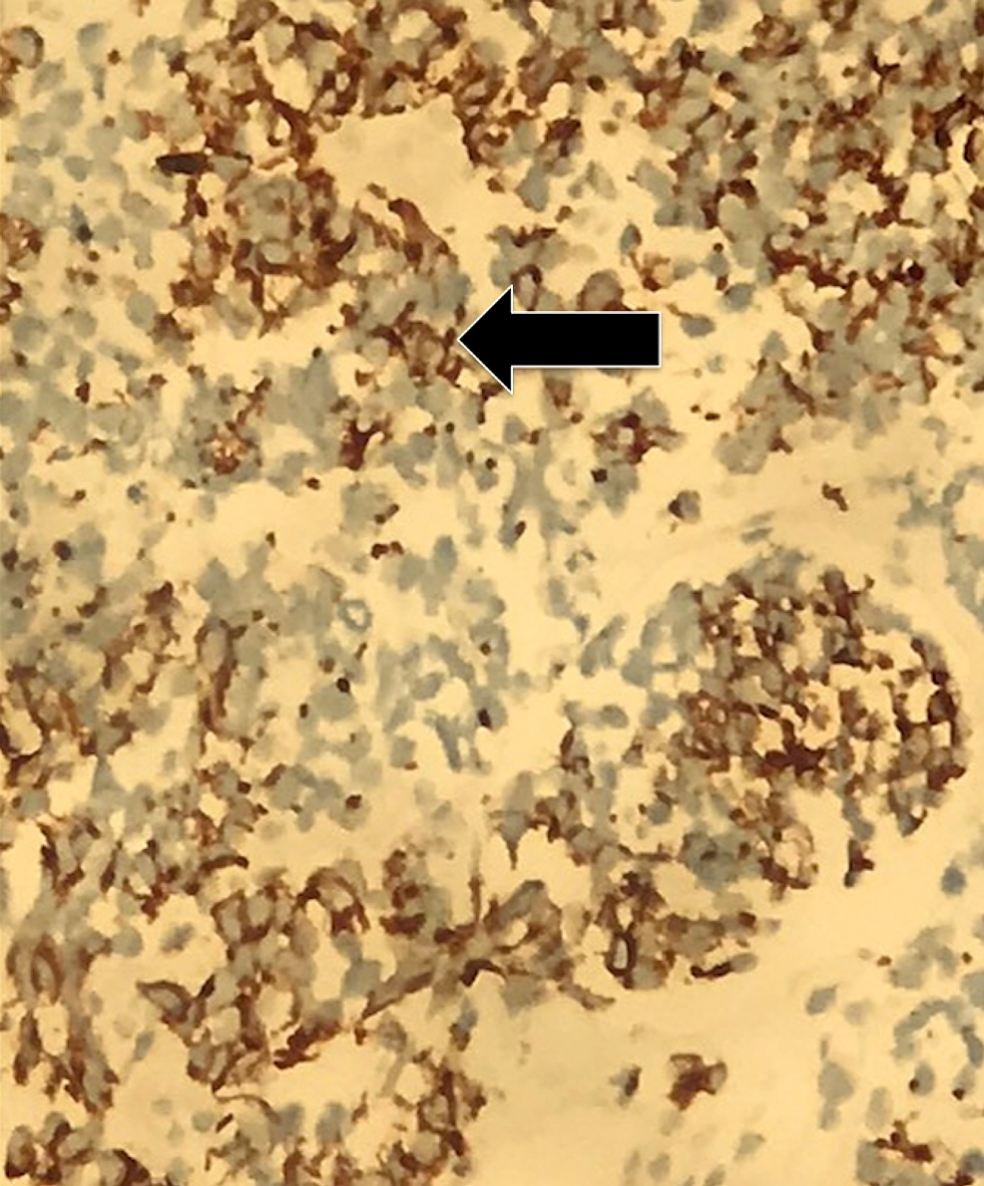
Pancytokeratin dot-like pattern positivity in neuroendocrine (arrow)

Histopathological analysis of tissue biopsied during EGD revealed gastric oxyntic mucosa with infiltrating mixed poorly differentiated adenocarcinoma (Figure 3) and neuroendocrine carcinoma (Figure 4). The uninvolved mucosa exhibited chronic gastritis with focal intestinal metaplasia. Immunostaining showed that the adenocarcinoma was diffusely and strongly positive for pancytokeratin (Figure 5), while the neuroendocrine component showed weaker dot-like staining (Figure 6).

**Figure 7:**
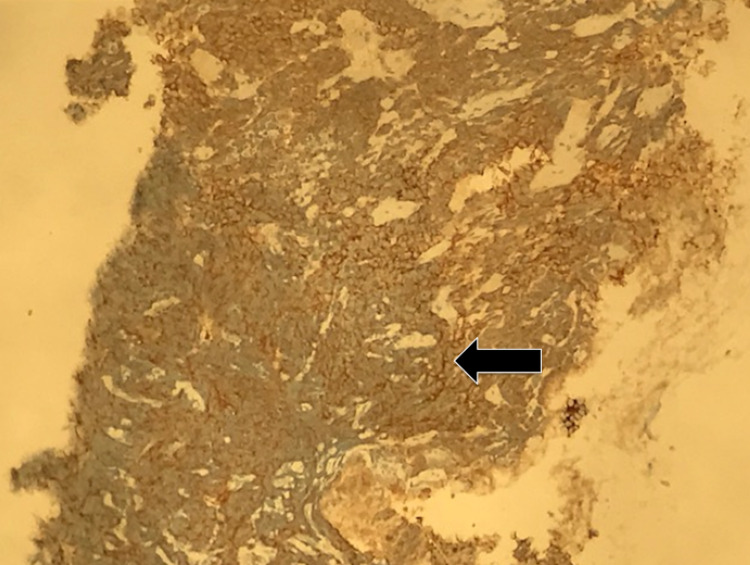
Synaptophysin diffuse positivity in neuroendocrine component (arrow)

**Figure 8:**
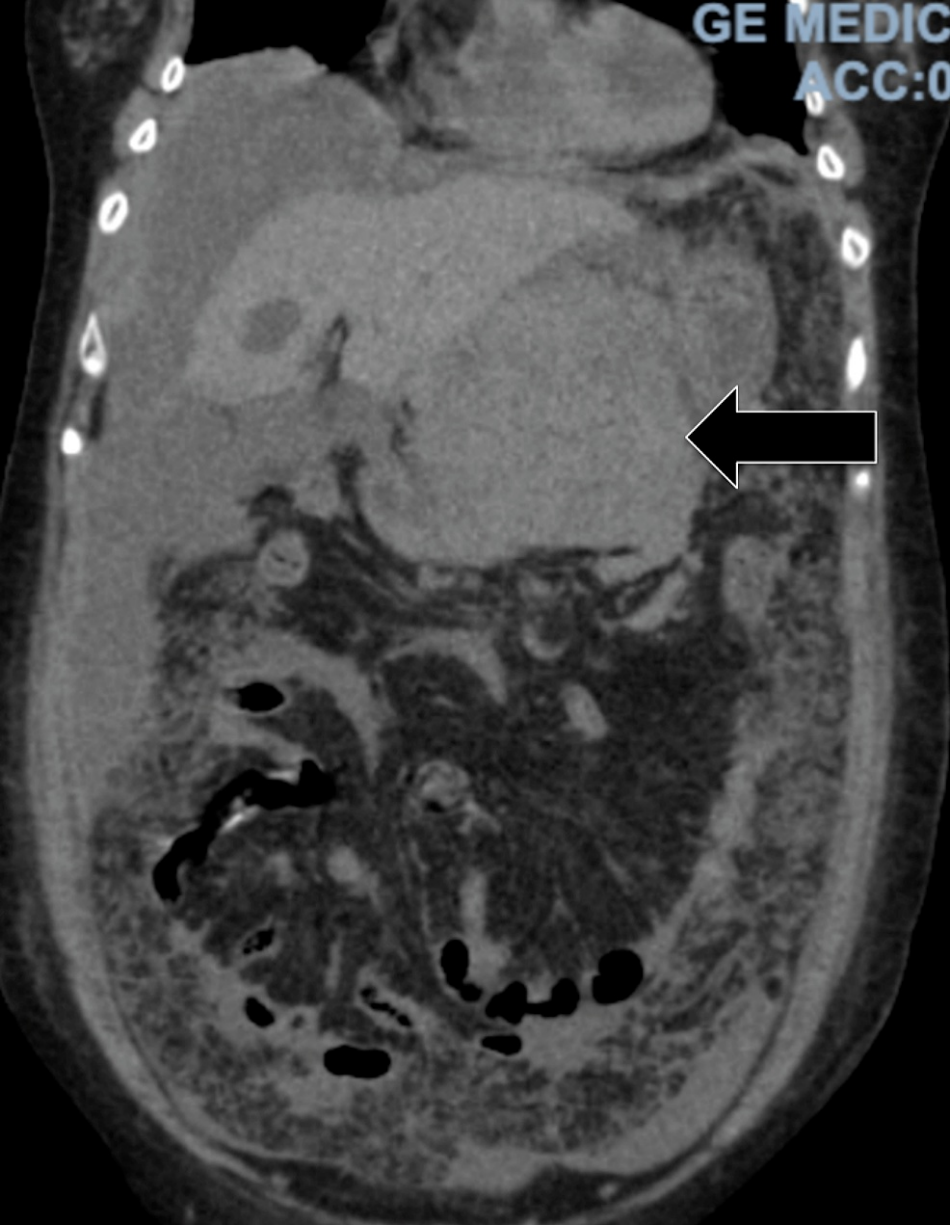
Repeat CT scan of the abdomen performed two weeks following admission The image shows an interval increase in the size of the mass extending from the stomach, as well as new metastatic liver lesions and moderate ascites (arrow) CT: computed tomography

The adenocarcinoma was positive for CDX2 while the neuroendocrine component was positive for synaptophysin and chromogranin (Figure 7). Immunostains for CD45 and *Helicobacter pylori* were negative. Alcian blue and periodic acid-Schiff staining highlighted many goblet cells. CT scans of the chest and abdomen were performed two weeks later, which revealed bilateral pulmonary embolism, as well an interval increase in the size of the gastric mass now to 7 x 12 x 7 cm with new liver metastasis and moderate ascites (Figure 8). MRI of the head was performed, which showed no intracranial pathology. The patient was determined to be a poor surgical candidate for resection due to advanced disease and was subsequently transitioned to hospice care.

## Discussion

Since the first case of MANEC tumor was described in 1924, there have been few reported cases in the medical literature. MANEC tumors have been widely described as various types of carcinomas including collision tumors, goblet cell carcinomas, and adenocarcinoid. While the precise pathophysiology of MANEC tumors remains unknown, there are two popular proposed pathophysiologic mechanisms. One mechanism suggests the presence of two distinct precursor cells, whereas the other suggests the mutation of a multipotential stem cell into the random cell lines. However, the exact pathophysiology is still unknown. The diagnosis of a MANEC tumor is performed through immunohistochemistry, as seen in our case. 

MANEC tumors are composed of adenocarcinoma and neuroendocrine carcinoma components, each of which comprises at least 30% of the lesion [2,3]. These tumors can be further divided into two categories based on the grade of malignancy: high vs. intermediate. Histologically, the neuroendocrine component bears similarity to both small cell and large cell neuroendocrine carcinoma of the lung. The small cell component is formed by small-sized cells with scanty cytoplasm and fusiform nuclei, while the large cell component is formed by cells with abundant cytoplasm and vesicular nuclei [3]. The non-neuroendocrine part of the MANEC tumors can be composed of either tubulovillous adenoma, villous adenoma, adenocarcinoma, or squamous cell carcinoma with the latter being very rare. From an immunohistochemical standpoint, the neuroendocrine component of these neoplasms can be positive for synaptophysin, chromogranin, or CD56. At least two of these markers must be present in order to diagnose a tumor as high-grade MANEC. MANEC tumors have been reported in different parts of GI tracts such as the esophagus and colon. Macroscopically, these neoplasms appear as polypoid and ulcerating masses and can grow up to a maximum diameter of 14 cm [3]. 

Our patient's tumor was composed of both neuroendocrine carcinoma and mixed poorly differentiated adenocarcinoma, with each component composing at least 30% of the cancer. Immunostaining of the biopsy showed that the adenocarcinoma was diffusely and strongly positive for pancytokeratin and CDX2. Immunostaining was also positive for synaptophysin and chromogranin, strongly indicative of MANEC. Immunostaining of the neuroendocrine carcinoma for pancytokeratin showed weaker dot-like staining. Our patient's tumor exhibited very aggressive characteristics with rapid expansion in size over the course of only two weeks, reaching nearly 13 cm in size. 

Due to the low incidence of these tumors, devising treatment plans can be challenging. If a tumor is noted to be predominantly an adenocarcinoma, treatment is geared toward surgery. A previous study including 80 patients showed that the three-year overall survival in patients with adenocarcinoma-dominant tumors was higher with lower recurrence rates compared to neuroendocrine-dominant tumors: 75% vs. 40% respectively, p=0.006 [4]. In the same study, it was also found that neuroendocrine-dominant tumors were an independent risk factor for worse overall survival [4]. In general, surgical resection is indicated in patients without metastasis and can be curative in <30% of patients; however, the recurrence rate is generally high [4,5]. Due to the high recurrence rate after surgery, chemotherapy has been the treatment of choice of patients, with median survival rates ranging from six to 12 months [4,6]. In our case, given the tumor size and since metastasis surgery was not an option, the patient was transitioned to comfort care. Given the rarity of the disease and the late presentation of most patients, there are no clear recommendations regarding chemotherapy regimens, and they are often left to the discretion of the treating physician.

## Conclusions

MANEC tumors are composed of both adenocarcinoma and neuroendocrine carcinoma components and are not well-described in the medical literature. Due to this scarcity of studies/data in the literature, the precise pathophysiology of the condition remains unclear, with various theories proposing different pathophysiological mechanisms. Diagnosis of a tumor as MANEC requires identifying specific histological features, such as the neoplasm exhibiting adenocarcinoma and neuroendocrine carcinoma components each comprising at least 30% of the lesion. IHC staining must show positivity for at least two of the three markers including synaptophysin, chromogranin, and CD56 in order to meet the criteria for diagnosis. MANEC tumors are often highly aggressive; however, a few reports have documented favorable outcomes with surgery or combined chemotherapy (cisplatin with irinotecan or etoposide) if diagnosed at an early stage.
